# Maple sap microbial communities and tubing sanitation: dominance of *Pseudomonas* species

**DOI:** 10.1128/mra.00693-25

**Published:** 2025-11-05

**Authors:** Olivia McHugh, Elijah Ayilaran, Yangjin Jung

**Affiliations:** 1 Agricultural & Environmental Research Station, West Virginia State University 2338, Institute, West Virginia, USA; Indiana University, Bloomington, Bloomington, Indiana, USA

**Keywords:** maple sap, microbial communities, tubing sanitation

## Abstract

Shotgun metagenomic data from maple (*Acer saccharum*) sap collected via 3/16-inch tubing (new, unsanitized, or sanitized with 400 ppm calcium hypochlorite) during weeks 1 and 5 revealed 317 species. Applying a 5% relative abundance cutoff, 20 dominant species were identified. *Pseudomonas_E proteolytica* and/or *Pseudomonas_E lurida* were abundant across conditions.

## ANNOUNCEMENT

Maple syrup is a natural sweetener produced by concentrating sap extracted from sugar maple trees (*Acer saccharum*). Plastic tubing systems are used to transport the sap from several trees into a collection reservoir, where it is concentrated through reverse osmosis and heating to become syrup (1). Microorganisms are known to colonize maple sap tubing systems and form biofilms within this tubing, which can adversely affect sap flow, color, flavor, and texture (2). Calcium hypochlorite (Ca(ClO)₂), empirically used to sanitize 3/16-inch tubing, was recently shown to be effective in lowering microbial load on tubing surfaces (3). However, its potential impact on the overall microbial community composition remains poorly understood (1, 3). To address this gap, 12 sap samples were collected from tubing lines at a maple sap research site in West Virginia (38.9°N, 79.2°W) with landowner permission during weeks 1 and 5 of the collection season. For each time point, two samples were obtained from each treatment group: 1-year-old non-sanitized (NS), new (N), and tubing sanitized (S) with 400 ppm Ca(ClO)₂. Metagenomic sequencing was performed to compare the microbial communities across the samples.

DNA was extracted from 50 mL of sap samples using the DNeasy Power Water Kit (Qiagen, Hilden, Germany) according to the manufacturer’s instructions. DNA quantification, library preparation, shotgun DNA sequencing, and taxonomic profiling were performed by CosmosID (Germantown, MD) as described previously (4). Briefly, DNA libraries were prepared using the Nextera XT DNA Library Preparation Kit (Illumina, San Diego, CA) with IDT Unique Dual Indexes (Integrated DNA Technologies, Coralville, IA), purified with AMPure XP beads (Beckman Coulter, Brea, CA), and quantified with the Qubit dsDNA HS Assay Kit (Thermo Fisher Scientific, Waltham, MA). Sequencing was conducted on the AVITI System (Element Biosciences, San Diego, CA) with the Cloudbreak 2 × 150 bp Kit (Element Biosciences). Default parameters were applied for all software unless otherwise specified.

For read processing, quality control was performed using FastQC (v0.11.9; Babraham Bioinformatics, Cambridge, UK); no additional cleaning or trimming was performed based on the FastQC results. For taxonomic profiling, Kepler Host-Agnostic Taxonomic Profiling was used in June 2024 (CosmosID Kepler v1.1.0; CosmosID Inc., Germantown, MD); high-quality reads were analyzed against the curated GenBook biomarker database using variable-length n-mer generation, exact k-mer matching, and Smith–Waterman refinement for taxonomic classification and abundance estimation (accessed via CosmosID-Hub, https://docs.cosmosid.com/docs/kepler-microbiome-profiler).

**Fig 1:**
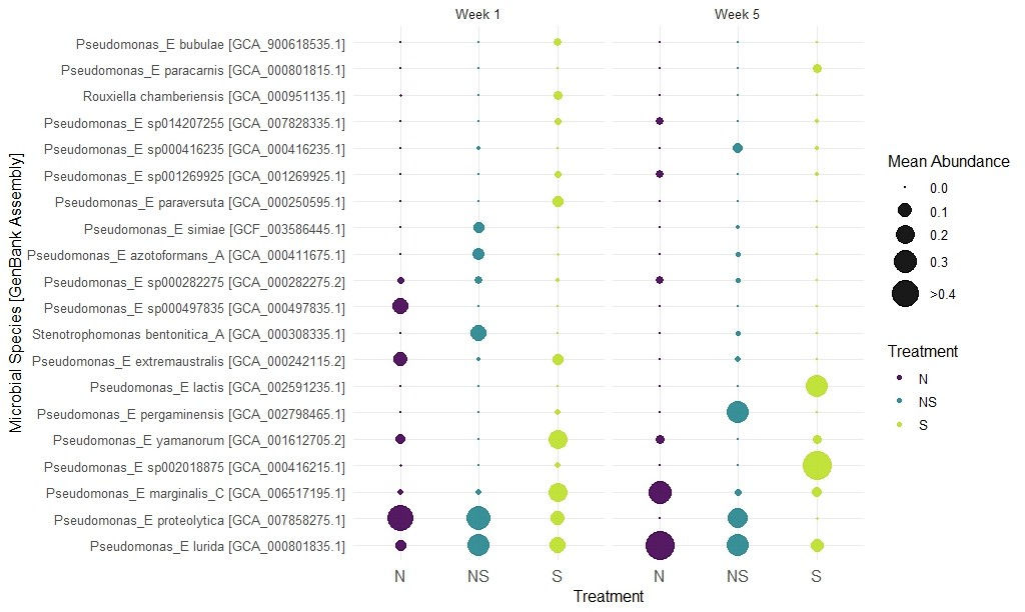
Relative abundance of microbial species (≥5%) and GenBank Accessions in maple sap samples collected from new (N), sanitized (S), and non-sanitized (NS) tubing at weeks 1 (W1) and 5 (W5). Circle size represents mean relative abundance.

Sequencing reads averaged 5.4 to 12.5 million per sample with a mean GC content of 59.2% (Table 1). In total, 6 phyla, 74 genera, and 317 species were identified across all samples. To assess the effects of tubing treatment (new, sanitized, and non-sanitized) and sap collection time (week 1 vs. week 5), PERMANOVA was conducted using the adonis2 function in the vegan package (R 4.4.2) based on Bray-Curtis dissimilarities of relative abundance data (5). No significant differences were found (*P* > 0.05). Consequently, species-level composition was visualized as a bubble plot (R 4.4.2, ggplot2) using a 5% relative-abundance threshold to highlight dominant taxa (Fig. 1). *Pseudomonas*_E *proteolytica* and/or *Pseudomonas*_E *lurida* were abundant (>5%) in most conditions.

**TABLE 1:** Accession numbers and characteristics of shotgun sequencing data of microbial species within sap collected from non-sanitized tubing, new tubing, and tubing sanitized with 400 ppm of Ca(ClO)_2_ solution

Sample	Time point	Tubing treatment	Total number of reads	GC (%)	Phred score	SRA accession
Y2-W1-N1	Week 1	New	5,889,990	60.4	38.6	SRX28109620
Y2-W1-N2	Week 1	New	7,114,736	59.9	41.9	SRX28109631
Y2-W1-NS1	Week 1	Non-sanitized^*a*^	10,877,452	59.8	40.3	SRX28109621
Y2-W1-NS2	Week 1	Non-sanitized	7,311,970	61.6	41.8	SRX28109627
Y2-W1-S1	Week 1	Sanitized	12,528,321	52.7	41.3	SRX28109624
Y2-W1-S2	Week 1	Sanitized	8,211,626	58.2	41.2	SRX28109629
Y2-W5-N1	Week 5	New	9,488,115	60.2	42.3	SRX28109625
Y2-W5-N2	Week 5	New	5,804,543	60.0	39.0	SRX28109626
Y2-W5-NS1	Week 5	Non-sanitized	5,798,271	58.4	41.8	SRX28109619
Y2-W5-NS2	Week 5	Non-sanitized	9,401,453	58.9	42.4	SRX28109628
Y2-W5-S1	Week 5	Sanitized	7,507,576	58.4	42.3	SRX28109622
Y2-W5-S2	Week 5	Sanitized	5,439,629	61.6	38.2	SRX28109623

^*a*^ Non-sanitized tubing denotes 1-year-old tubing that was reused from the prior sap collection season without undergoing any sanitation treatment.

## Data Availability

The raw sequence reads generated in this study have been deposited in the NCBI Sequence Read Archive (SRA) under the accession number PRJNA1240310. The data set includes 12 SRA experiments and associated BioSample records. Table 1 provides the SRA accession numbers along with direct links to each data set.

## References

[1] Lagacé L, Jacques M, Mafu AA, Roy D. 2006. Compositions of maple sap microflora and collection system biofilms evaluated by scanning electron microscopy and denaturing gradient gel electrophoresis. Int J Food Microbiol 109:9–18. doi:10.1016/j.ijfoodmicro.2006.01.004 16515815

[2] N’guyen GQ, Roblet C, Lagacé L, Filteau M. 2022. A metataxonomic analysis of maple sap microbial communities reveals new insights into maple syrup complexity. Front Syst Biol 2:893007. doi:10.3389/fsysb.2022.893007

[3] Jung Y, McHugh O, Ayilaran E. 2024. Application of calcium hypochlorite for sanitizing 3/16-inch tubing used in maple sap collection. Microorganisms 12:10. doi:10.3390/microorganisms12101948 PMC11509703 39458258

[4] Liu L, Firrman JA, Narrowe AB, Mahalak KK, Lemons JMS, Marzorati M, Duysburgh C, Rotsaert C, Van de Wiele T. 2025. Structural and functional characterization of a porcine intestinal microbial ecosystem developed in vitro. Sci Rep 15:24821. doi:10.1038/s41598-025-10144-5 40640502 PMC12246222

[5] Zhang Y, Jewett C, Gilley J, Bartelt-Hunt SL, Snow DD, Hodges L, Li X. 2018. Microbial communities in the rhizosphere and the root of lettuce as affected by Salmonella-contaminated irrigation water. FEMS Microbiol Ecol 94:fiy135. doi:10.1093/femsec/fiy135 30010741

